# Low prevalence of the molecular markers of *Plasmodium falciparum* resistance to chloroquine and sulphadoxine/pyrimethamine in asymptomatic children in Northern Benin

**DOI:** 10.1186/1475-2875-12-413

**Published:** 2013-11-13

**Authors:** Aurore Ogouyèmi-Hounto, Nicaise Tuikue Ndam, Gildas Fadégnon, Carmine Azagnandji, Mourchidath Bello, Azizath Moussiliou, Jean-Phillipe Chippaux, Dorothée Kinde Gazard, Achille Massougbodji

**Affiliations:** 1 Unité d’Enseignement et de Recherche en Parasitologie Mycologie de la Faculté des Sciences de la Santé 01 BP188, Bénin; 2 Laboratoire du Centre de Lutte Intégrée contre le Paludisme, Cotonou 01 BP188, Bénin; 3 PRES Sorbonne Paris Cité, Faculté de Pharmacie, Université Paris Descartes, France; 4 Institut de Recherche pour le Développement, UMR216 Mère et enfant face aux infections tropicales, Cotonou 08 BP 841, Bénin

**Keywords:** *Plasmodium falciparum*, Genotyping, Resistance, Mutation, Chloroquine, Sulphadoxine-pyrimethamine

## Abstract

**Background:**

In Benin, very few studies have been done on the genetics of *Plasmodium falciparum* and the resistance markers of anti-malarial drugs, while malaria treatment policy changed in 2004. Chloroquine (CQ) and sulphadoxine pyrimethamine (SP) have been removed and replaced by artemisinin-combination therapy (ACT). The objective of this study was to determine the genetic diversity of *P. falciparum* and the prevalence of *P. falciparum* molecular markers that are associated with resistance to CQ and SP in northern Benin seven years after the new policy was instituted.

**Methods:**

The study was conducted in northern Benin, a region characterized by a seasonal malaria transmission. Blood samples were collected in 2012 from children presenting with asymptomatic *P. falciparum* infections. Samples collected in filter paper were genotyped by primary and nested PCR in block 2 of *msp-1* and block 3 of *msp-2* to analyse the diversity of *P. falciparum.* The prevalence of critical point mutations in the genes of *Pfcrt* (codon 76), *Pfmdr1* (codon 86), *Pfdhfr* (codons, 51, 59 and 108) and *Pfdhps* (codons 437, 540) was examined in parasite isolates by mutation-specific restriction enzyme digestion.

**Results:**

Genotyping of 195 isolates from asymptomatic children showed 34 *msp-1* and 38 *msp-2* genotypes. The multiplicity of infection was 4.51 ± 0.35 for *msp-1* and 4.84 ± 0.30 for *msp-2.* Only the codon 51 of *Pfdhfr* and codon 437 of *Pfdhps* showed a high mutation rate: I51: 64.4% (57.3; 71.2); G437: 47.4% (40.2; 54.7), respectively. The prevalence of *Pfdhfr* triple mutant IRN (I51, R59 and N108) was 1.5% (0.3; 3.9), and *Pfdhfr/Pfdhps* quadruple mutant IRNG (*Pfdhfr*I51, R59, N108, and *Pfdhps*G437): 0. 5% (0; 2.5). No mutation was found with codon 540 of *Pfdhps*. Analysis of mutation according to age (younger or older than ten years) showed similar frequencies in each category without significant difference between the two groups.

**Conclusions:**

This study showed a high diversity of *P. falciparum* in northern Benin with a very low prevalence of resistance markers to CQ and SP that dramatically contrasted with the pattern observed in southern Benin. No influence of age on genetic diversity of *P. falciparum* and on distribution of the mutations was observed.

## Background

Malaria is one of the killer diseases in the world and particularly in sub-Saharan Africa where 90% of deaths due to malaria are recorded [1]. In Benin, *Plasmodium falciparum* infections are among the leading causes of disease and are also 36% of cause of death among children under five years (unpublished data from Ministry of Health). Despite intensification of control methods against malaria, multiple factors, including insecticide resistance in anopheline vectors and the emergence and rapid spread of drug-resistant strains, remain of major concern in efforts to control and prevent malaria. In this context, adequate vaccine development is a big challenge in malaria control. However, this approach is complicated by genetic diversity of *P. falciparum* as it influences the acquisition of protective immunity to malaria. Asexual blood stage antigens, such as merozoite surface protein-1 (*msp-1*) and merozoite surface protein-2 (*msp-2*) are considered prime candidates for the development of malaria vaccine and are also suitable markers for the identification of genetically distinct *P. falciparum* parasite subpopulations [2]. These two genes are also the basis for determining the multiplicity of infection (MOI) in infected individuals, which is a good indicator of acquired immunity or premunition of populations living in endemic areas, and is also correlated to transmission intensity [3,4].

Following the change of malaria policy treatment in Benin in 2004 with the replacement of chloroquine (CQ) and sulphadoxine pyrimethamine (SP), the first- and second-line treatment, by artemisinin combination therapy (ACT) for the treatment of uncomplicated malaria, drug pressure with these molecules has since been reduced. Unlike in Malawi [5,6], a study performed in southern Benin [7] revealed high rates of resistant genotypes in genes P*fcrt, Pfmdr1, Pfdhfr, and Pfdhps*. Although SP is still used in intermittent preventive treatment in pregnancy (IPTp) as recommended by WHO, recent observation of such high rates of resistant parasite mutants suggested that despite the official withdrawal of CQ and SP from the treatment of uncomplicated malaria, these drugs were still used in Benin.

Parakou is a semi-urban city situated in the north of the Republic of Benin and has been well characterized as a highly endemic area with seasonal malaria transmission. However, there is no information on the genetic diversity of *P. falciparum* populations regarding resistance markers in this area. The present study was conducted in this locality to determine: (i) the genetic diversity of *P. falciparum* based on the *msp-1* and *msp-2* polymorphism; and, (ii) the prevalence of *P. falciparum* molecular markers that are associated with resistance to CQ and SP by analysing the point mutations in *Pfcrt, Pfmdr1*, *Pfdhfr and Pfdhps* gene using samples from asymptomatic children in northern Benin.

## Methods

### Study sites and population

The study was conducted in the city of Parakou, a municipality in the north of Benin. It is the largest semi-urban city in northern Benin with 188,853 inhabitants. In the north, transmission occurrs from June to October during the rainy season and is spread by *Anopheles gambiae s.s*. (85%) and *Anopheles arabiensis* (15%) [8]. Children aged two to 15 years, asymptomatic and permanent residents of the study area, were enrolled from May to September 2012 from nursery and primary schools. The recruitment of these children has been described elsewhere [7]. Inclusion criteria were: i) axillary temperature < 37.5°C; ii) absence of fever in the previous two weeks and at least one week after enrolment; and, iii) positive thick smear regardless of parasite density.

### Collection and handling of blood samples

Thick and thin blood smears were prepared from venous blood, stained with 10% Giemsa for rapid diagnosis and were examined against 500 leucocytes. Parasite densities were recorded as the number of parasites/μl of blood assuming an average leucocyte count of 8,000/μl of blood. For samples containing parasites, four separate drops of blood were spotted and stored on to Whatman 3 filter paper for further DNA extraction. All slides were read in the health centre’s laboratory with external quality control of 10% of the negatives slides and all positives in the Reference Laboratory of Parasitology of the Centre National Hospitalier Universitaire of Cotonou. Suspected malaria patients, following microscopy results, were treated according to malaria treatment policy based on ACT: artemether-lumefantrine. Parasite DNA was extracted from filter papers using the Chelex100 resin methods [9] and stored at −20°C until use.

### Molecular genotyping of the polymorphic genes msp-1 and msp-2

Specific primer pairs were used to amplify block 2 of *msp-1* and block 3 of *msp-2*[10,11]. The two genes were amplified by primary and nested PCR, each amplification with conserved or family-specific primer pairs being done separately, as described previously [12]. Analysis of the 3D7 and FC27 allelic families of *msp-2* and the K1, MAD20 and RO33 allelic families of *msp-1* were sequentially performed in accordance with the genotyping protocol of Snounou *et al.*[12]. Allelic-specific positive controls and DNA-free negative controls were included in each set of reaction. Gel photographs were re-scored by visual comparison of DNA fragments and for individual samples, alleles were identified according to band size and the corresponding allele-specific primers used. The size of the PCR products was estimated using a 100 bp DNA ladder marker (Boehringer Mannheim, Marker VI).

### PCR amplification of Pfcrt, Pfmdr, Pfdhfr and Pfdhps genes and detection of Single nucleotide polymorphisms (SNPs)

Parasite DNA was amplified with outer and nested specific primers targeting the *Pfcrt, Pfmdr1*, *Pfdhfr* and *Pfdhps* genes, as described [13-15]. The Single nucleotide polymorphisms in the various gene targets were detected using the Restriction Fragment Length Polymorphism (RFLP) technique previously reported by Ogouyemi Hounto *et al.*[7]. The molecular analyses were performed in the Molecular Biology Laboratory of the Centre de Lutte integrée contre le Paludisme.

### Data analysis

The data were entered in the software R version 2.12.0 (R Foundation for Statistical Computing, Vienna, Austria). The distribution of allelic families of *msp-1 and msp-2* genes was determined by the number of PCR products corresponding to each family within the total number of samples. The number of patients with more than one amplified PCR fragment within the total population is defined as the frequency of polyclonal infections. The MOI was determined as the number of different *msp-1* and *msp-2* genotypes per isolate, and the mean MOI was calculated as the total number of detected *P. falciparum msp-1, msp-2* genotypes/total number of infected children [16]. Each codon was characterized as wild type (no mutation present), pure mutant (only mutant genotypes detected). Cases of mixed infection (wild type and mutant) were categorized as mutant throughout the analysis. The frequency of a particular mutant was calculated as the proportion of the specific mutant samples among the total number of samples successfully analysed for this mutation. Similarly, the frequencies of double, triple and quadruple mutants were determined as the proportion of subjects with two, three and four mutations among the total numbers of samples tested for the each. To investigate the relationship between the mutation, MOI and age, children were segregated into two categories: children below and above ten years of age. This grouping resulted from recent intensification of malaria control activities in the country, such as the widespread distribution and use of insecticide-treated nets and large-scale indoor spraying of residual insecticides, which are likely to delay the age of acquisition of immunity, usually occurring at five years in endemic areas [17]. In areas where malaria transmission is seasonal, the acquisition of immunity is later than in the areas with perennial transmission. Student’s test was used to compare MOI. The Chi-square test or Fisher’s exact test was used for proportion comparisons. The p value <0.05 was chosen as threshold significance for the various statistical tests.

### Ethical approval

This study obtained the ethical approval of the National Ethics Committee for Health Research of Benin.

## Results

### Demographic and parasitological data of the study population

During five months, 1,642 asymptomatic children were screened, from which 214 positive samples for *P. falciparum* were collected on filter paper. The prevalence of asymptomatic parasitaemia detected by microscopy was 13%, *P. falciparum* being the only infecting *Plasmodium* species found. After the withdrawal of children above 15, 195 samples were selected for molecular analysis.

Characteristics of the study population are detailed in Table 1. Children’s ages ranged from two to 15 years (mean age: 7.9 ± 0.4 years). The parasite density ranged from 12 to 86,570 parasites/μl with a geometric mean density of 209.6 (161.2-272.5).

**Table 1:** Demographic and parasitological data of the study population

**Characteristics of patients**	**Values**
Mean age (year)	7.9 ± 0.4
Age range (year)	2-15
Sex ratio (M/F)	1.03 (99/96)
Microscopy *P. falciparum* prevalence, 214/1,642	13%
Geometric mean parasite density (p/μl)	209.6 (161.2-272.5)
Parasite density range (p/μl)	12-86,570

The parasite DNA from the 195 *P. falciparum* isolates were analysed for *msp-1* and *msp-2* genes. The efficiency of *msp-1* and *msp-2* genes amplification reactions with family-specific primers was 99% (193/195) and 99.5% (194/195), respectively.

Regarding the analysis of the mutation, the number of isolates depended on the success of PCR amplification and was 195 for *Pfcrt* (100%), 193 (99%) for *Pfmdr1*, 192 (98.5%) for *Pfdhps* and 194 (99.5%) for *Pfdhfr*. Samples that were retested to check the reproducibility of results were consistent with those found initially.

### Genetic diversity of *Plasmodium falciparum* msp- 1 and msp-2 gene

A total of 72 individual *msp* alleles were identified (34 for *msp-1* and 38 for *msp-2*). The K1 family was the predominant allelic type among monoclonal infections (17.1%), in mixed infection with Mad20 (13.5%) and RO33 (11.9%). This family was also more represented (p < 0.01) in overall population (90.7%). Mad20 and Ro33 families were found respectively in 67.4 and 66.8% of isolates. For *msp-2* gene, the FC27 family was more represented but not statistically significant (p = 0.57). The distribution of the different allelic families of *msp-1* and *msp-2* genes is shown in Additional file 1: Table S1.

The number of *msp-1* and *msp-2* genotypes per isolate ranged from one to 11. MOI was 4.51 ± 0.35 for *msp-1* and 4.8 ± 0.30 for *msp-2.* According to age, MOI was 4.8 ± 0.43 in children below ten and 4.1 ± 0.59 in older children with *msp-1* gene (p = 0.06). Regarding *msp-2* gene, MOI was 4.3 ± 0.46 and 3.9 ± 0.60, respectively, in children below ten and older children, respectively, p = 0.18. Multiple infections were found in 71.5% (138/193) for *msp-1* and 91.7% (178/194) for *msp-2*.

### Prevalence of Pfcrt and Pfmdr1, alleles and mutations

**Figure 1:**
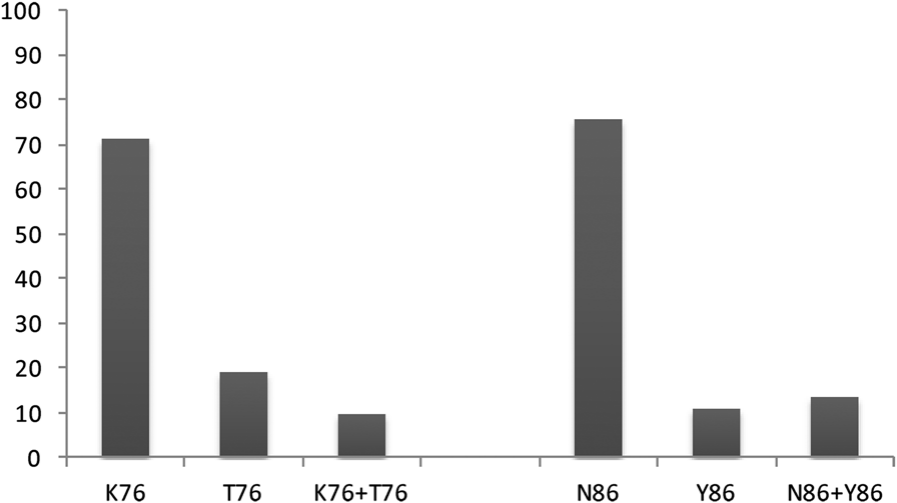
**Prevalence of P** ***fcrt *** **and *Pfmdr1 *** **alleles.**

The wild type alleles K76 and N86 of *Pfcrt* and *Pfmdr1* gene, respectively, were present in 71.3% (139/195) and 75.6% (146/193) of samples from patients that were analysed, whereas mutant and mixed alleles were poorly represented (Figure 1). Thus, prevalence of T76 mutation was 28.7% (56/193) (22.5; 35.6), while Y86 was found in 24.4% (47/193) (18.5; 31.0) (Table 2).

**Table 2:** **Prevalence of molecular markers associated with *Plasmodium falciparum *** **resistance to chloroquine and sulphadoxine-pyrimethamine in study population according to age**

**Molecular marker**	**n (%)**	**<10 years**	**≥10 years**	**P value**
		**n (%)**	**n (%)**	
T76 (n = 195)	56 (28.7)	38 (31.1)	18 (24.7)	0.42
Y86 (n = 193)	47 (24.4)	26 (22)	21 (28)	0.44
T76Y86 (n = 193)	10 (4.6)	6 (4.4)	4 (4.8)	1.00
G437 (n = 192)	91 (47.4)	56 (47.5)	35 (47.3)	0.90
E540 (n = 192)	0 (0)	0 (0)	0 (0)	
I51 (n = 194)	125 (64.4)	76 (61.8)	49 (69)	0.39
R59 (n = 194)	50 (25.8)	29 (24.2)	21 (28.4)	0.63
N108 (n = 194)	35 (18)	24 (20.2)	11 (14.7)	0.44
IRN (n = 194)	3 (1.5)	1 (0.7)	2 (2.4)	0.56
IRNG (192)	1 (0.5)	0	1 (1.2)	0.38

### Prevalence of Pfdhps and Pfdhfr allelic forms

#### *Pfdhps*

**Figure 2:**
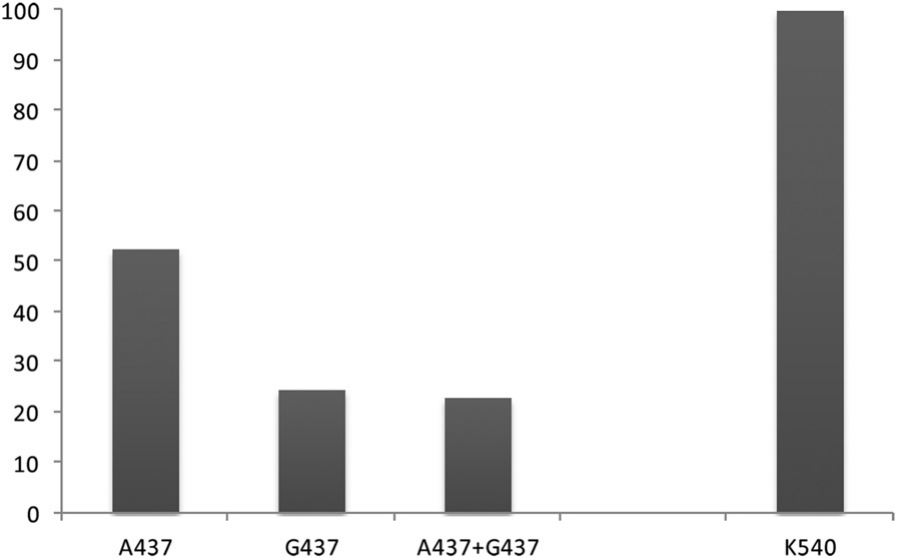
**Prevalence of *pfdhps *** **alleles.**

At codon 437, the wild type allele A437 (52.6%: 101/192) was more represented in the population than mutant (G437: 24.5%: 47/192) and mixture of alleles (A437/G437: 22.9%: 44/192), P < 0.01. However, all samples carried the wild type allele K540 of codon 540 and no mutation E540 was found (Figure 2, Table 2). Overall, mutation of codon 437 (G437) was found in 47.4% (40.2; 54.7) of the samples analysed (91/192).

#### *Pfdhfr*

**Figure 3:**
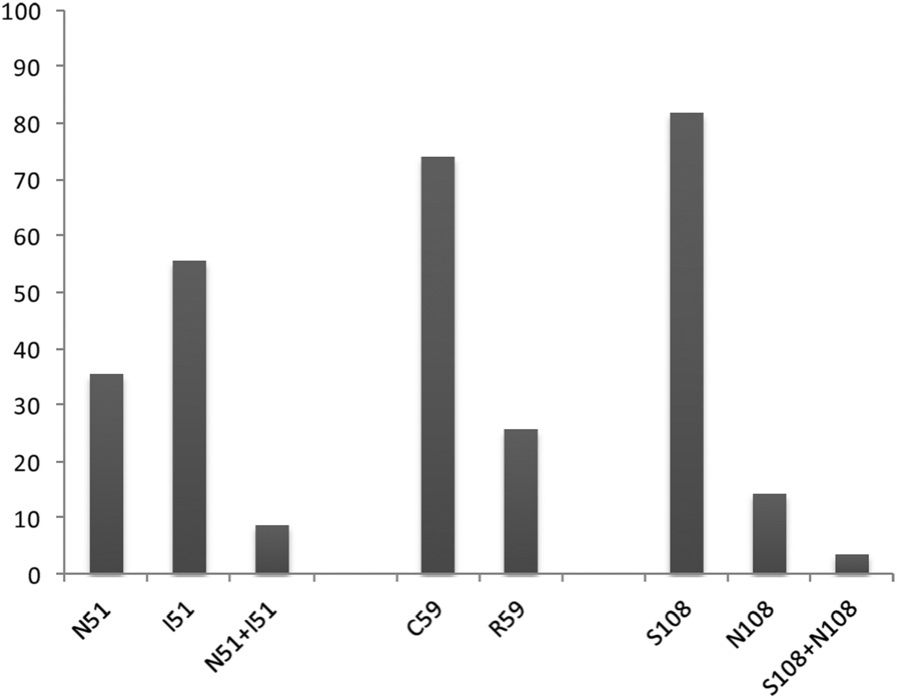
**Prevalence of *Pfdhfr *** **alleles.**

Except for codon 51 in which mutated alleles were more represented (I51: 55,7%: 108/194), the analysis of other codons showed high prevalence of wild type alleles. Allele C59 were found in 74.2% of the samples (144/194) and S108 in 82% (159/194) (Figure 3). Thus, a high prevalence of mutant genotypes was observed only at codon 51 (I51: 64.4% (125/194) (57.3; 71.2)) compared to other codons where the prevalence of mutants remained very low (R59: 25.8% (50/194) (19.8; 32.5), N108: 18% (35/194) (12.9; 24.2)) (p < 0.01). Combined analysis of the different Single nucleotide polymorphisms revealed that the prevalence of *Pfdhfr* triple mutant IRN (I51, R59 and N108) and *Pfdhfr/Pfdhps* quadruple mutant IRNG (*Pfdhfr* I51, R59, N108, and *Pfdhps*G437) was actually very low: IRN: 1.5% (3/194) (0.3; 3.9), IRNG: 0.5% (1/192) (0; 2.5) (Table 2).

### Mutation and age

When the data were analysed by age categories, similar frequencies of single, triple and quadruple mutant parasite were found in children younger than ten years and in older children (Table 2).

### Mutation and polyclonality

The analysis showed similar frequency of single, double, triple and quadruple mutation in monoclonal and polyclonal infections regardless of the gene (*msp-1* or *msp-2*). However, the mutation G437 seemed to be more represented in polyclonal infections of *msp-1* gene (Table 3).

**Table 3:** Mutation and polyclonality

**Mutations**	***Msp-1 *** **(n = 193)**			***Msp-2 *** **(n = 194)**		
	**Monoclones**	**Polyclones**	**p-value**	**Monoclones**	**Polyclones**	**p-value**
	**n (%)**	**n (%)**		**n (%)**	**n (%)**	
	n = 55	n = 138		n = 16	n = 178	
	n (%)	n (%)		n (%)	n (%)	
T76	19 (34.5)	37 (26.8)	0.10	6 (37.5)	50 (28.1)	0.39
Y86	9 (16.4)	38 (27.5)	0.40	1 (6.3)	46 (25.8)	0.23
T76Y86	5 (9.1)	5 (3.6)	0.13	0	10 (5.6)	0.78
G437	14 (25.5)	77 (55.8)	0.01	6 (37.5)	85 (47.7)	0.96
I51	31 (56.4)	94 (68.1)	0.48	8 (50.0)	117 (65.7)	0.77
R59	15 (27.3)	35 (25.4)	0.44	2 (12.5)	48 (27)	0.49
N108	10 (18.2)	25 (18.1)	0.74	1 (6.3)	34 (19.1)	0.46
IRN	2 (3.6)	1 (0.7)	0.07	0	3 (1.7)	1.00
IRNG	1 (1.8)	0	0.56	0	1 (0.6)	1.00

## Discussion

In the Republic of Benin, less attention had been put on the investigation of the genetic diversity of *P. falciparum* and on the molecular markers of *P. falciparum* resistance to CQ and SP since the change of malaria treatment policy in 2004. The purpose of this study was to determine the genetic diversity of *P. falciparum* using the two most polymorphic regions of *msp-1* and *msp-2* genes, and the prevalence of molecular markers of *P. falciparum* to CQ and SP in malaria asymptomatic subjects in northern Benin. This analysis intends to develop efficient strategies for malaria control, and improve the surveillance of the actual level of *P. falciparum* resistance to anti-malarial drugs in Benin.

This study follows a previous one that was conducted in southern Benin [7]. Allele-specific genotyping of *msp-1* and *msp-2* showed a high genetic diversity in the *P. falciparum* population studied in Parakou with a MOI of 4.5 *(msp-1*) and 4.8 (*msp-2*). This figure is similar to that observed in southern Bénin (unpublished data). Actually, a minimum of 34 alleles of *msp-1* were observed, in which the K1 allelic family was predominant, consistent with the previous study [18-21] but in contrast to Barty [22] in India showing that RO33 family was predominant. For the *msp-2* locus, 38 alleles were found and alleles belonging to FC27 family were mostly detected, both in mono-infection and mixed infection with 3D7 alleles. Although this is similar to data reported in Congo [23], it differs from previous results of Issifou *et al.* in south Benin [19], and Mayengue *et al.* in Congo Brazzaville [24]. The difference with the results of Issifou *et al.* may be due to the fact that the study goes far back in the past. Indeed, according Yuang *et al.* in Myanmar [25], the majority of alleles showed significant temporal fluctuations through the years. The high rate of multiple infections of *P. falciparum* infections with *msp-1* and *msp-2* found elsewhere [16,26] would probably be a consequence of intense malaria transmission of study areas. Actually, the mean MOI was high compared to those reported in Benin, Burkina Faso, Congo Brazzaville [18,19,24], but consistent those from Gabon and Senegal [20,27]. The high rate of MOI in the present study suggests that, despite the intensification of malaria control interventions involving reduction of malaria infections, the parasite population size and transmission intensity remained high enough to allow effective genetic recombination of the parasites and continued maintenance of genetic diversity. The fact that MOI was not influenced by age as shown in other countries [28,29] suggests that the MOI is not directly related to the period of acquisition of immunity in asymptomatic children, but reflects the exposure of subjects to malaria in endemic area.

The major finding of this study is that analysis of well-characterized molecular markers of *P. falciparum* resistance to CQ and SP, two anti-malarials that have long been used in the treatment of uncomplicated malaria in Benin, revealed contrasted low prevalence of resistant genotypes in northern Benin as opposed to the south [7]. A low prevalence of T76 (28.7%) mutation associated with resistance to CQ was observed in this study compared to the study carried out in the south in 2011 where a high prevalence of this mutation was observed, indicating a persistence of resistance to CQ seven years after the change of malaria treatment policy. The same goes for the quadruple mutation associated with resistance to SP, which is very low in this setting compared to data reported in southern Benin. The dramatic difference between the results could be explained by a higher drug pressure in southern Benin. Parakou is a semi-urban city 407 km from Cotonou in the north of the country where self-medication and a parallel market for counterfeit drugs are lower than in the south, where proximity to Nigeria promotes drug traffic and pressure. Duah *et al.* in Ghana [30] observed an increase of drug-resistant genotypes in the urban setting compared to rural areas. Some studies [31,32] have shown the selection of sensitive phenotypes of *Pfcrt* and *Pfmdr1* gene after stopping the use of CQ and extending the use of the AL. Data in southern Benin where the policy change was made at the same time does not show this reversion and still is an extremely high prevalence of *pfcrt* and *Pfmdr1* mutants [7]. However, the likely effect of the introduction of artemether-lumefantrine in the explanation of this phenomenon, which may be even more pronounced in areas where the pressure is lower like north Benin can be taken into account.

The migration of parasites related to population movements between the north and south of Benin could increase the prevalence of resistant parasites. However the inclusion of children living in Parakou for at least six months has probably allowed the minimizing of such a risk. This does not preclude transmission of resistant parasites by mosquito bites on persons in transit harbouring resistant parasites. Certainly the magnitude of this phenomenon is minimal and not enough to significantly increase the proportion of resistant parasites.

As malaria treatment policy was harmonized in the country when policy change was decided in 2004, it had not been taken into account places where low rates of treatment failure had been noted. Indeed, in Parakou in 2002, *in vivo* efficacy studies conducted according to WHO protocol [33] reported 13.9% of treatment failure with CQ (4.6 and 9.3% early and late treatment failures, respectively), which was lower compared to the national average of 35.2% treatment failure (unpublished data from NMCP of Ministry of Health). Based on the association between treatment failure to CQ and the prevalence of T76 mutation [13,34], the results in this study rather suggest the existence of a small proportion of mutation T76 before the policy change. This also suggests that the low prevalence observed in this study is unlikely to result from the re-emergence of susceptible strains related to the reduction of drug pressure.

Regarding SP, the therapeutic failure rates observed in 2002 in Parakou were 3.3% (1.6 and 1.7% early and late treatment failures, respectively) against the 22.8% national average. Given the relation between triple *Pfdhfr* mutations, double *Pfdhps* mutations and clearance of parasitaemia after SP treatment [35-37], the very low rates of parasite with quadruple *Pfdhfr Pfdhps* mutant (IRNG: 0.5%) found in this study, suggests that there was very little parasite resistance to SP in the study area before the withdrawal of the drug from the treatment of uncomplicated malaria in 2004. In this study, the high mutation rates on codon 51 quite surprising, which almost twice the level in the codon 108 mutation was observed. There is no logical explanation for this, but in some studies mutation rates of codon 51 and 108 appeared to be substantially the same as described by Dicko et *al*. [38], and the selection of mutants could be faster with the codon 51. It is likely that the use of SP in IPTp did not contribute to an increase in the rate of resistant parasites, in agreement with studies reported in Senegal and Mali [38,39]. The data generated in this study suggest that these two molecules could still be used in the treatment of malaria in Parakou, however, the difficulty in policy management of malaria in the country argues for a harmonized strategy.

The proportion of infections by parasites carrying specific mutations between children younger than ten years and older children suggests that age does not influence the distribution and carriage of resistant parasites whatever the type of mutation, as shown elsewhere [7,40]. To investigate whether the mutation patterns were influenced by polyclonality, the parasite isolates were classified as monoclonal and polyclonal. Apart from the G437 mutation, which was more prevalent in polyclonal infections, all other mutations: single, double, triple or quadruple, were distributed similarly in both groups regardless of the gene. These results suggest that the number of clones is not directly involved in the carriage of mutant parasites*.* However, it would be interesting to investigate the link between polyclonality and mutation in a population with high prevalence of mutation in order to draw better conclusions.

## Conclusions

This study showed a high diversity of *P. falciparum* in northern Benin irrespective of age in asymptomatic children. It also highlighted a low prevalence of markers of parasite resistance to CQ and SP, indicating that most *P. falciparum* strains are still potentially sensitive to these molecules in this part of the country. These results strongly opposed to those in the south which shows the need to map molecular markers of resistance in different regions of endemic countries, taking into account the drug pressure.

### Consent

All parents or guardians of children gave their informed consent to participate in the study and the possible use of the data for publication.

## Competing interests

The authors declare that they have no competing interests.

## Authors’ contributions

DKZ designed the study protocol, supervised the study and corrected the manuscript; AOH participated in the design of study, supervised the study and laboratory examinations, drafted the manuscript; NTN supervised laboratory examinations, monitored laboratory quality assurance and participated in manuscript writing; GF, CA, MB, and AM assured patients’ enrolment, molecular analysis and participated in manuscript writing; JPC coordinated and helped to draft the manuscript. AM participated in the design of the study, coordination and helped to draft the manuscript. All authors read and approved the final manuscript.

## Supplementary Material

Additional file 1: Table S1 **Genetic diversity of *P. falciparum msp-1 and msp-*** **2 gene.** Click here for file
