# Supplementary material for: Low prevalence of the molecular markers of Plasmodium falciparum resistance to chloroquine and sulphadoxine/pyrimethamine in asymptomatic children in Northern Benin
Source: Malar J. 2013 Nov 13;12:413. doi: 10.1186/1475-2875-12-413 (PMC3834525; doi:10.1186/1475-2875-12-413)
Supplement: Additional file 1: Table S1 — Genetic diversity of P. falciparum msp-1 and msp- 2 gene. [file 1475-2875-12-413-S1.docx]

**Genetic diversity of *P. falciparum msp-1 and msp-*2 gene**

| Family | N (%) | Family | N (%) |  |
| --- | --- | --- | --- | --- |
| **MSP1** | n= 193 | **MSP2** | N=194 |  |
| K1 | 33 (17) | 3D7 | 6 (3.1) |  |
| MAD20 | 4 (2.1) | FC27 | 20 (10.3) |  |
| RO33 | 8 (4.1) | 3D7+FC27 | 168 (86.6) |  |
| K1 + MAD20 | 26 (13.5) | Total 3D7 | 174 (89.7) |  |
| K1 + RO33 | 23 (11.9) | Total FC27 | 188 (96.9) |  |
| MAD20 + RO33 | 6 (3.1) | Multiplicity of infection | 4.8 ±0.30 |  |
| K1 + MAD20 + RO33 | 93 (48.2) | Polyclonal infections | 178 (91.7) |  |
| Total K1 | 175 (90.7) |  |  |  |
| Total Ro33 | 130 (67.4) |  |  |  |
| Total Mad20 | 129 (66.8) |  |  |  |
| Multiplicity of infection | 4.5 ±0.35 |  |  |  |
| Polyclonal infections | 138 (71.5) |  |  |  |
